# Cellular Level *In-silico* Modeling of Blood Rheology with An Improved Material Model for Red Blood Cells

**DOI:** 10.3389/fphys.2017.00563

**Published:** 2017-08-02

**Authors:** Gábor Závodszky, Britt van Rooij, Victor Azizi, Alfons Hoekstra

**Affiliations:** ^1^Computational Science Lab, Faculty of Science, Institute for Informatics, University of Amsterdam Amsterdam, Netherlands; ^2^Department of Hydrodynamic Systems, Budapest University of Technology and Economics Budapest, Hungary; ^3^ITMO University Saint Petersburg, Russia

**Keywords:** blood rheology, RBC material model, cellular flow, high-performance computing, dense cell initialization

## Abstract

Many of the intriguing properties of blood originate from its cellular nature. Therefore, accurate modeling of blood flow related phenomena requires a description of the dynamics at the level of individual cells. This, however, presents several computational challenges that can only be addressed by high performance computing. We present Hemocell, a parallel computing framework which implements validated mechanical models for red blood cells and is capable of reproducing the emergent transport characteristics of such a complex cellular system. It is computationally capable of handling large domain sizes, thus it is able to bridge the cell-based micro-scale and macroscopic domains. We introduce a new material model for resolving the mechanical responses of red blood cell membranes under various flow conditions and compare it with a well established model. Our new constitutive model has similar accuracy under relaxed flow conditions, however, it performs better for shear rates over 1,500 *s*^−1^. We also introduce a new method to generate randomized initial conditions for dense mixtures of different cell types free of initial positioning artifacts.

## 1. Introduction

On the cellular level, blood is a dense suspension of various types of cells. Red blood cells (RBC) form the primary component with an approximate volume fraction of 42% (Davies and Morris, 1993) determining the bulk blood rheology. They have a biconcave shape and a typical diameter of 8 μ*m*. Platelets (PLTs), the second most numerous component with typically 1 PLT for every 10 RBCs (Björkman, 1959) form the link between transport dynamics and vital biochemical processes related to thrombus formation. In their unactivated state PLTs have a rigid ellipsoidal form. The collective behavior of RBCs and PLTs can provide explanation to the most fundamental transport phenomena in blood, for instance the non-Newtonian viscosity (Merrill and Pelletier, 1967), the margination of platelets (Beck and Eckstein, 1980; Tilles and Eckstein, 1987), the Fåhræus effect (Barbee and Cokelet, 1971), the appearance of a cell-free layer (Maeda et al., 1996; Kim et al., 2009), or the scaling of shear-induced diffusion of RBCs (Mountrakis et al., 2016). The necessity to accurately reproduce these effects grows as the typical length-scale of the examined system reaches below ≈ 200 μ*m*, at which point the macroscopic description no longer yields accurate local dynamics (Popel and Johnson, 2005). With the development of modern medical devices more and more elements reside in the micrometer domain, such as the strut structure of flow-diverters (Lubicz et al., 2010) or woven endobridge (WEB) flow disruptor devices (Ding et al., 2011). This together with additional complex phenomena that require detailed cellular modeling of the flow, for instance platelet aggregation (Nesbitt et al., 2009) or white blood cell (WBC) trafficking (Fay et al., 2016), triggers an increasing need to understand how the rheology and the transport of the RBCs and PLTs are influenced while acting over such small scales.

In the solutions targeting these questions the mechanical responses of the RBCs and PLTs are often expressed with constitutive models applied through their membranes accounting for the responses of the various structural elements (Ye et al., 2016). Some examples for these material models are the spectrin-link membrane model of Dao et al. (2006) or the energy model of Skalak et al. (1973). Fedosov et al. (2010a) employed the dissipative particle dynamics (DPD) method with a constitutive description gained by coarse-graining the model of Dao et al. (2006) to study various transport features of blood (Fedosov et al., 2010b, 2011b,c; Fedosov and Gompper, 2014; Yazdani and Karniadakis, 2016). A low dimensional RBC membrane model was developed by Pan et al. (2010) and was compared to the coarse-grained spectrin-link model of Fedosov et al. (2010a). More recently, a two-component RBC membrane model that consist of a separate lipid bilayer and spectrin network (Chang et al., 2016) was introduced to examine the difference in the deformation of healthy and infected RBCs. MacMeccan et al. (2009) developed a model that coupled the lattice Boltzmann method (LBM) to finite element method (FEM) based cell mechanics and investigated the viscosity behavior of blood in shear flows at various hematocrit levels. Later, Reasor et al. (2012) used the spectrin-link membrane representation rather than solving Cauchy's equation to model the trajectory and deformation of elastic deformable particles. This model was also used to study the margination of platelets (Mehrabadi et al., 2016). Moreover, Krüger et al. (2011) developed a combination of lattice Boltzmann method (LBM) and finite element RBC membrane model based on the energy model of Skalak et al. (1973), and used the immersed boundary method (IBM) to couple the fluid and the membrane. This model was used to study the tank-treading behavior of single RBCs next to the deformation behavior and the relative viscosity of RBC suspensions (Krüger et al., 2013; Gross et al., 2014; Krüger, 2016). In addition, Shi et al. used LBM in combination with the fictitious-domain method to couple the plasma to the spectrin-link membrane model. They studied the deformation of an RBC in capillary flows, during tank-treading motion and hydrodynamic interaction between two cells (Shi et al., 2014). Hashemi and Rahnama (2016) investigated the deformation of RBCs in capillary flows with an LBM-FEM based model with IBM coupling.

In this paper, our framework called Hemocell (High pErformance MicrOscopic CELlular Libary)^1^ is presented for modeling the flow of blood on a cellular level. Hemocell is designed to be easily extendible with additional cell-types and interactions and to provide the high computational performance that enables applications up to macroscopic scales. Blood plasma is represented as a continuous fluid simulated with LBM, while the cells are represented as discrete element method (DEM) membranes coupled to the fluid flow by the immersed boundary method. Furthermore, two different material models for the RBC membrane mechanics have been investigated. One is the aforementioned coarse-grained spectrin-link model of Fedosov et al. (2010a) and a new one that addresses several shortcomings of the former. The validation of Hemocell, in combination with our new RBC material model, is presented through single-cell mechanical experiments (i.e., stretching and shearing cases). We demonstrate that the proposed new material model reproduces both the single-cell mechanical responses and the collective transport dynamics in very good agreement with experiments, as well as it provides an accurate mechanical response and an increased structural stability under higher shear forces and strong deformations. The later is necessary, since it is known from recent high-field-strength MRI measurements of Bouvy et al. (2016) that pulsation effects are significant even on the mesoscopic level of smaller arterioles. Moreover, it can also enable simulations of transport mechanisms in micro-fluidic settings or in the vicinity of micro-medical devices, where strong deformations and high shear values and gradients can be expected. Hemocell also forms a fundamental component in building versatile multi-scale models of arterial health and diseases (Hoekstra et al., 2016).

## 2. Methods

**Figure 1:**
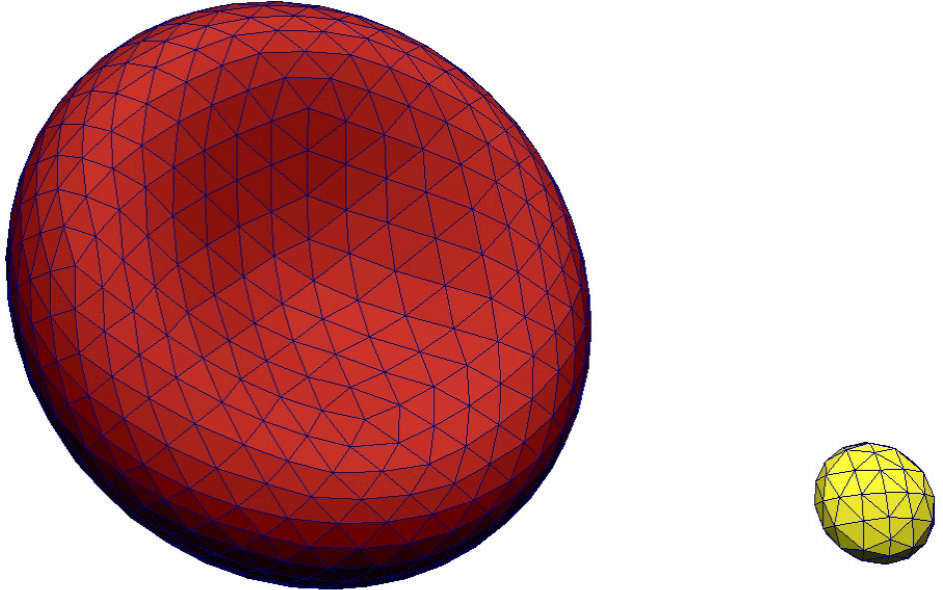
Visualization of the membrane meshes of an RBC **(Left)** and a platelet **(Right)**.

The solvent (blood plasma) in Hemocell is modeled as an incompressible Newtonian fluid using the lattice Boltzmann method implemented in the Palabos library (Lagrava et al., 2012) which is known to be capable of producing accurate flow results in vascular settings (Závodszky and Paál, 2013; Anzai et al., 2014). The surfaces of RBCs and PLTs are are described as boundary layers immersed into the plasma. These layers are discretized using *N*_*v*_ vertices which are connected by *N*_*e*_ edges yielding *N*_*t*_ surface triangles (see Figure 1 for an example in case of an RBC and a PLT). The connectivity and symmetries are similar to the structure of the cytoskeleton as imaged by atomic force microscopy (Swihart et al., 2001; Liu et al., 2003). In our simulations the membrane of each RBC consisted of *N*_*v*_ = 642 vertices, *N*_*e*_ = 1, 920 edges, and *N*_*t*_ = 1, 280 faces. The mechanical behavior of a cell is expressed using this discrete membrane structure. The response to deformations is formulated as a set of forces acting on the cell membrane, which is coupled to the plasma flow through a validated in-house immersed-boundary implementation (Mountrakis et al., 2014; Mountrakis, 2015) that has an efficient parallel design. Mountrakis et al. (2015) demonstrated that the framework can be scaled up to 10^6^ cells executing on 8,192 cores without significant loss of parallel efficiency.

### 2.1. Description of the coarse-grained spectrin-link membrane model

In Hemocell, two distinct constitutive model have been implemented for RBCs to act on the membrane mesh. The first one is based on the systematic coarse-graining of the model of Dao et al. (2006). For the detailed derivation we refer to the work of Fedosov et al. (2010a). The model is briefly outlined below:

The free-energy of a cell is described as

$$U_{total}=U_{in-plane}+U_{bend}+U_{volume}+U_{area}+U_{visc}.$$

The location *x*_*i*_ of each vertex on the membrane mesh is updated according to the force:

$$F_{i}=\frac{\partial U_{total,i}}{\partial x_{i}}.$$

The total free-energy is composed of the following elements:

1. The in-plane potential models the compression response of the underlying cytoskeletal network along the membrane surface. The edges of the surface triangles represent the cumulative behavior of the local spectrin links using the wormlike chain (WLC) nonlinear spring description:
  $$U_{in-plane}=\underset{i=1..N_{e}}{\sum }U_{WLC}+\underset{j=1..N_{t}}{\sum }\frac{C_{q}}{A_{k}^{q}},$$
  $$U_{WLC}=\frac{k_{B}Tl_{m}}{4p}\frac{3r_{i}^{2}-2r_{i}^{3}}{1-r_{i}},$$
  $$C_{q}=\frac{\sqrt{3}A_{l_{0}}^{2}k_{B}T\left(4r_{0}^{2}-9r_{0}+6\right)}{4pl_{m}{\left(1-r_{0}\right)}^{2}},$$
  where *p, l*_*m*_ are the persistence length and the maximum length of the spectrin links, *r*_*i*_ = *l*_*i*_/*l*_*m*_ ∈ [0, 1), *l*_0_ is the average length of links, *r*_0_ = *l*_0_/*l*_*m*_ and $A_{l_{0}}=\sqrt{3}l_{0}^{2}/4$.
2. The potential to account for bending rigidity:
  $$U_{bend}=\underset{i=1..N_{e}}{\sum }\tilde{\kappa }\left[1-cos\left(\theta_{i}-\theta_{0}\right)\right],$$
  where $\tilde{\kappa }=2\kappa /\sqrt{3}$, θ_*i*_ is the instantaneous, θ_0_ is the equilibrium angle between neighboring faces sharing an edge, and κ is the bending constant.
3. The volume conservation energy is a fictitious potential which accounts for the forces arising from the change of volume:
  $$U_{volume}=\frac{k_{V}{\left(V-V_{0}\right)}^{2}}{2V_{0}},$$
  where *V* is the current, and *V*_0_ is the equilibrium volume of the cell.
4. The area conservation potential is similarly a non-physical term representing the inextensible nature of the bilipid layer:
  $$U_{area}=\frac{k_{A}{\left(A-A_{0}\right)}^{2}}{2A_{0}}+\underset{k=1..N_{t}}{\sum }\frac{k_{A_{l}}{\left(A_{k}-A_{0,k}\right)}^{2}}{2A_{0,k}},$$
  where *A, A*_0_ are the global and *A*_*k*_, *A*_0,*k*_ are the local actual and equilibrium surface areas, respectively.
5. The additional term to correct membrane viscosity:
  $$U_{visc}=\underset{i=1..N_{e}}{\sum }-\frac{1}{2}\eta_{m}v_{m,n}^{2},$$
  where *v*_*m,n*_ denotes the relative velocity of the vertices *m* and *n* connected by edge *i* and membrane viscosity $\eta_{m}=22\times 10^{-3}Pas$ is chosen such that RBCs yield realistic tank-treading and tumbling frequencies (Fedosov et al., 2014).

The free parameters of this model (κ = 100 *k*_*B*_*T*, *k*_*V*_ = 6, 000, *k*_*A*_ = 5, 900, *k*_*A*_*l*__ = 100) were adopted from (Fedosov et al., 2010a) with the exception of the maximum link extension ratio ($r_{0}=\frac{l_{0}}{l_{m}ax}=2.6$), which was fine-tuned for our current discretization. The usefulness of this model was demonstrated in a series of publications (Fedosov et al., 2010a, 2011a; Fedosov and Gompper, 2014; Mehrabadi et al., 2016). However, it also has a few shortcomings. The bending response is based on Helfrich's model (Helfrich, 1973) which only accounts for the properties of the lipid bilayer and not the underlying structures. Furthermore, the coarse-graining of the bending rigidity for the triangulated mesh is based on the work of Gompper and Kroll (1996) which assumes small angles and equilateral triangles, both of which are often not fulfilled for sheared RBCs. As a consequence, the bending energy in this model yields a sinusoidal force-response that has a sub-linear response for angles over $\frac{\pi }{4}$, which even decays further for larger angles. This can lead to insufficient force responses and consequently to acute angles or collapse of neighboring faces. The resulting problems can often be mitigated by using a linear bending response that fits the slope of the sinusoidal at low angles. Moreover, the global surface conservation potential can lead to unphysical responses, since a local stretch of the membrane instantly causes the contraction of the rest of the membrane forcing the surface points to move toward the center of the cell.

### 2.2. Description of the new constitutive model

We propose a new material model in the form of a set of forces acting on the same triangulated cellular membrane. The initial assumption for this model is that during small deformations all these forces present a linear regime with different slopes as the response types correspond to different components of the cell and are independent of each other. However, for large enough deformations the cytoskeleton adds contribution to all of them, resulting in qualitatively similar behavior. For instance, a response for small bending is assumed to be dominated by the curvature rigidity of the bilipid membrane resulting in a term linear in angle for the DEM membrane, while for larger deformation the underlying cytoskeleton deforms as well yielding an additional quickly diverging term. In the following we describe this model in two steps by separating the phenomenological description and the implementation.

1. The link force acts along links between surface points and represents the response to stretching and compression of the underlying spectrin-network beneath the representative link. The formulation of the force is similar in spirit to the worm-like-chain potential model often used to mimic the mechanical properties of polypeptide chains. It presents a linear part which corresponds to smaller deformations and a fast-diverging non-linear part which represents the limits of the material toward this type of deformation by quickly increasing the force response as the stretch approaches the persistence-length.
  $$F_{link}=-\frac{\kappa_{l}dL}{p}\left[1+\frac{1}{\tau_{l}^{2}-dL^{2}}\right],$$
  where $dL=\frac{L_{i}-L_{0}}{L_{0}}$ is the normal strain defined as the relative deviation from the equilibrium length *L*_0_ with τ_*l*_ = 3.0 is chosen based on the assumption that the represented spectrin-network reaches its persistence length at the relative expansion ratio of 3. The persistence-length of a spectrin filament was taken as *p* = 7.5 *nm* (Li et al., 2005).
2. The bending force acts between two neighboring surface elements representing the bending response of the membrane arising primarily from the non-zero thickness of the spectrin-network. On each surface it points along the normal direction of that surface. As opposed to the previous model in which bending is expressed by modeling the bending rigidity of the bilipid membrane (Helfrich, 1973), the form of the employed force term here is similar to the form of the previous link force to account for increased resistances coming for additional sources, such as the connection of the membrane to the underlying cytoskeleton.
  $$F_{bend}=-\frac{\kappa_{b}d\theta }{L_{0}}\left[1+\frac{1}{{\tau_{b}}^{2}-d\theta^{2}}\right],$$
  where *d*θ = θ_*i*_ − θ_0_. From simple geometric considerations it follows that the limiting angle τ_*b*_ scales with the discretization length of the surface elements (*L*_0_). We fix the smallest representable curvature $r_{min}=\frac{L_{0}}{2}\sin\frac{\tau_{b}}{2}$. From the micro pipette aspiration images of Mohandas and Evans (1994) a rough approximation for the necessary curvature radius of 0.2 μ*m* can be inferred by examining the membrane curvature at the pipette neck. For the currently employed resolution (*L*_0_ = 0.5 μ*m*) the limiting angle is chosen to be $\tau_{b}=\frac{\pi }{6}$. This choice prevents unrealistic sharp surface edges while allowing curvature radii as small as 0.18 μ*m* to be represented.
3. The local surface conservation force acts locally on surface elements (i.e., triangles) and has the same form. It represents the combined surface response of the supporting spectrin-network and the lipid bilayer of the membrane to stretching and compression. This force is applied to all three vertices of each face and it points toward the centroid of the corresponding surface triangle.
  $$F_{area}=-\frac{\kappa_{a}dA}{L_{0}}\left[1+\frac{1}{{\tau_{a}}^{2}-dA^{2}}\right],$$
  where $dA=\frac{A_{i}-A_{0}}{A_{0}}$. Strong-deformation experiments of erythrocytes show that at around 40% of surface area change the membrane of most cells is damaged permanently (Li et al., 2013). We set τ_*a*_ = 0.3, thus prohibiting surface area changes larger than 30%.
4. The volume conservation force is the only global term. It is used to maintain quasi-incompressibility of the cell. It is applied at each node of each surface element and it points toward the normal of the surface.
  $$F_{volume}=-\frac{\kappa_{v}dV}{L_{0}}\left[\frac{1}{\tau_{v}^{2}-dV^{2}}\right],$$
  where $dV=\frac{V-V_{0}}{V_{0}}$, τ_*v*_ = 0.01 and κ_*v*_ = 20 *k*_*B*_*T* is chosen to be a large but numerically still stable constant.

This constitutive model has three free parameters for RBC modeling : κ_*l*_, κ_*b*_, and κ_*a*_. These are chosen to satisfy mechanical single-cell experimental results.

### 2.3. Implementation of the constitutive forces for the new model

**Figure 2:**
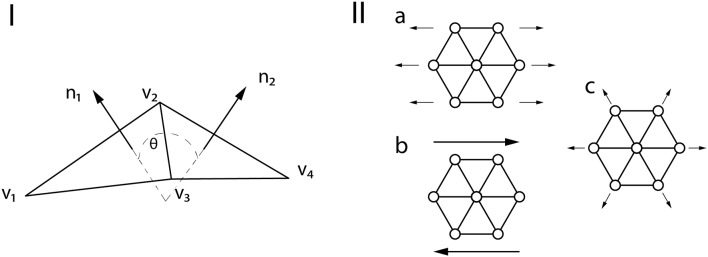
**(I)** Notation of the membrane structure. **(II)** Mechanical tests on a patch of membrane. **(IIa)** uniaxial stretching. **(IIb)** shearing. **(IIc)** area expansion.

The proposed forces can be realized in multiple ways on the given DEM structure, thus the implementation method is an inseparable part of the model. Figure 2I aids this description by showing a notation for two neighboring surface elements.

1. For each edge ${\vec{e}}_{i},i\in \left[1..N_{e}\right]$ the link force ${\vec{F}}_{link}$ is added to the total force acting on the end nodes of that edge (i.e., the IBM particles). Following the notation of Figure 2I for the edge between the nodes ${\vec{v}}_{1}$ and ${\vec{v}}_{2}$, the resulting link forces are:
  $${\vec{F}}_{link_{v_{1}}}=F_{link}*\frac{{\vec{v}}_{2}-{\vec{v}}_{1}}{\left||{\vec{v}}_{2}-{\vec{v}}_{1}|\right|}=-{\vec{F}}_{link_{v_{2}}}.$$
2. The bending force is applied for each edge ${\vec{e}}_{i},i\in \left[1..N_{e}\right]$ on the four nodes of the two connecting surface elements. For the edge between the nodes ${\vec{v}}_{1}$ and ${\vec{v}}_{3}$:
  $${\vec{F}}_{bend_{v_{k}}}=-F_{bend}*{\vec{n}}_{k},k\in \left[1,2\right]$$
  $${\vec{F}}_{bend_{v_{l}}}=F_{bend}*\frac{{\vec{n}}_{1}+{\vec{n}}_{2}}{2},l\in \left[3,4\right].$$
3. The local surface conservation force acts on each ${\vec{f}}_{j},j\in \left[1..N_{t}\right]$ surface elements. For the face with the normal vector ${\vec{n}}_{1}$ and centroid $\vec{C}$:
  $${\vec{F}}_{area_{v_{m}}}=F_{area}*\left(\vec{C}-{\vec{v}}_{m}\right),m\in \left[1,2,3\right].$$
4. The volume conservation force is applied on the three nodes of each surface element:
  $${\vec{F}}_{volume_{j}}=F_{volume}*\frac{S_{j}}{S_{avg}}*{\vec{n}}_{j},$$
  where *S*_*j*_ is the surface area of the j-th element and *S*_*avg*_ is the average surface area.

### 2.4. Validation of the mechanical RBC responses

**Figure 3:**
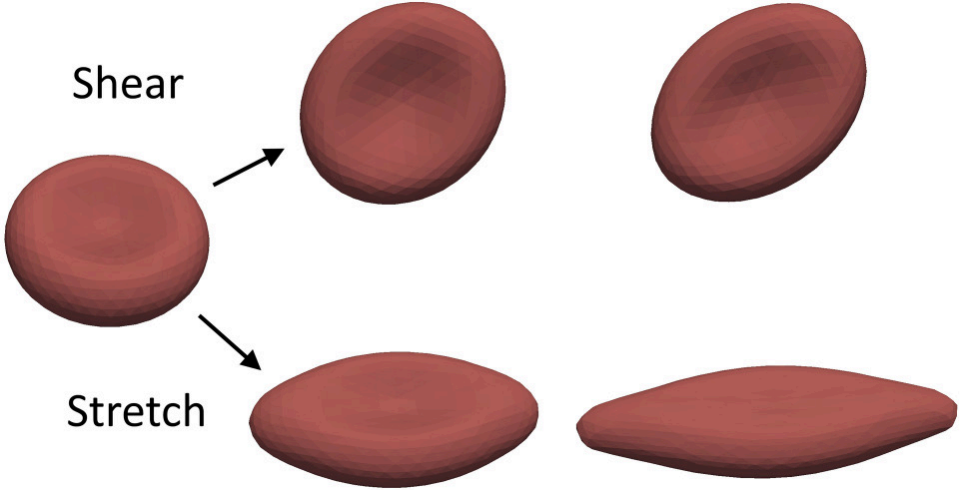
The mechanical properties of the RBCs are validated using experiments measuring these two basic deformations.

The free parameters of our mechanical RBC membrane model are fit to match the results of the optical-tweezer stretching experiments (Mills et al., 2004) and the Wheeler shear experiment (Yao et al., 2001). The single-cell deformation types during the measurements are shown in Figure 3.

In the optical-tweezers experiment small silica beads are attached on the opposing sides of the RBC. One is then fixed to the wall of the experimental container while the other is moved away by a focused laser beam. The arising forces result in a stretching of the RBC along the longitudinal axis and contraction along the transversal axes. In our simulation the same force magnitudes are used as in the experiment. They are applied on five percent of the membrane area on the opposing ends of the RBC. These areas represent the attachment surfaces of the silica beads.

**Figure 4:**
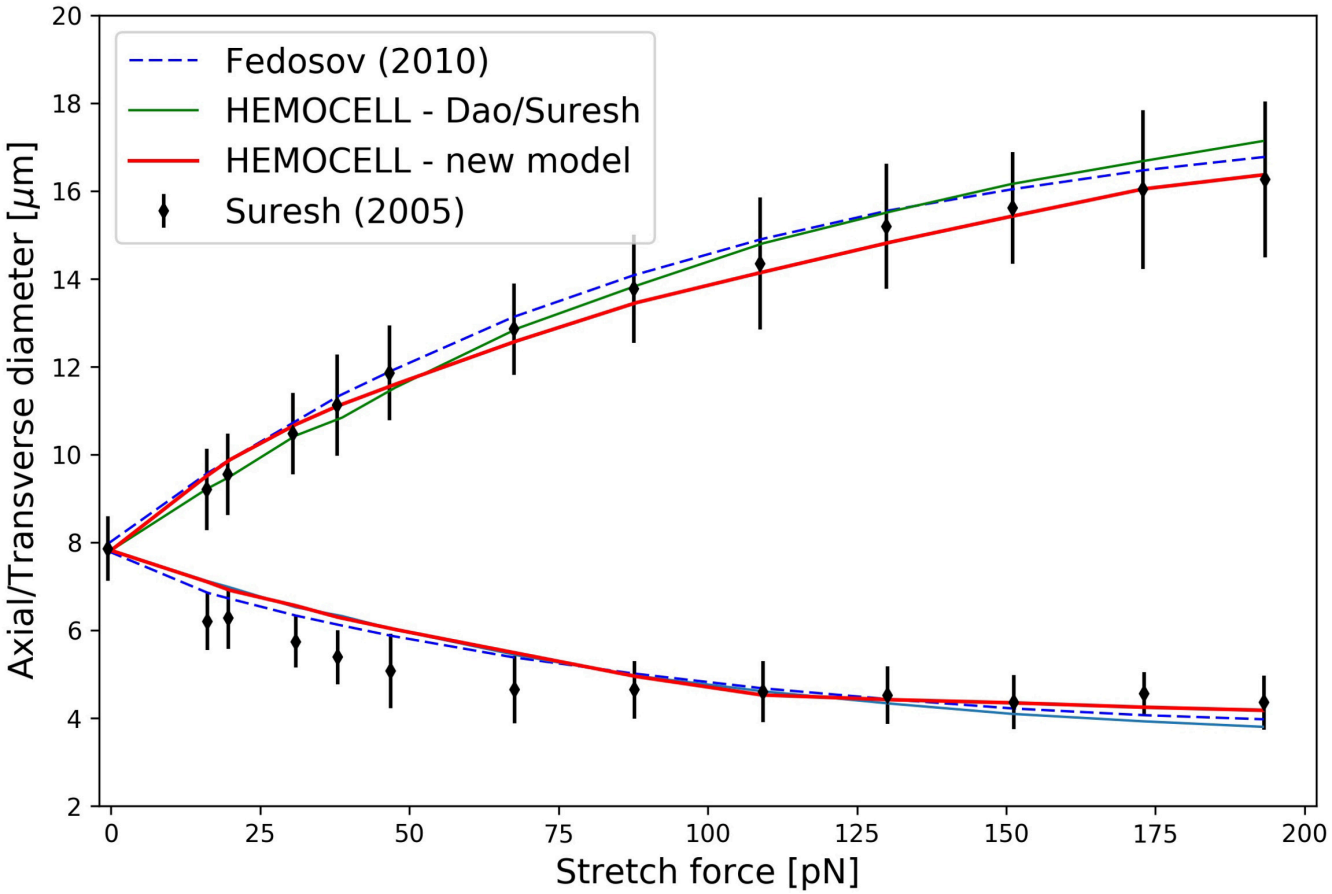
The results of the RBC stretching simulations. The upper arm of the curve denotes axial diameter expansion due to the stretching force, while the lower arm depicts the transversal contraction of the cell.

The stretching curves of the two material models implemented in Hemocell are compared to the experiment of Mills et al. (2004) and to the results of Fedosov et al. (2010a) and are shown in Figure 4. Both constitutive membrane models can reproduce the stretching behavior of a single RBC in the given forcing regime with good accuracy. However, since the responses of the different force types are more balanced in our new model (i.e., it is less likely, that one force will dominate over the others during deformation), while the spectrin-link model is over-dominated by the in-plane link-force, our model captures the transversal contraction at higher stretches with more accuracy.

**Figure 5:**
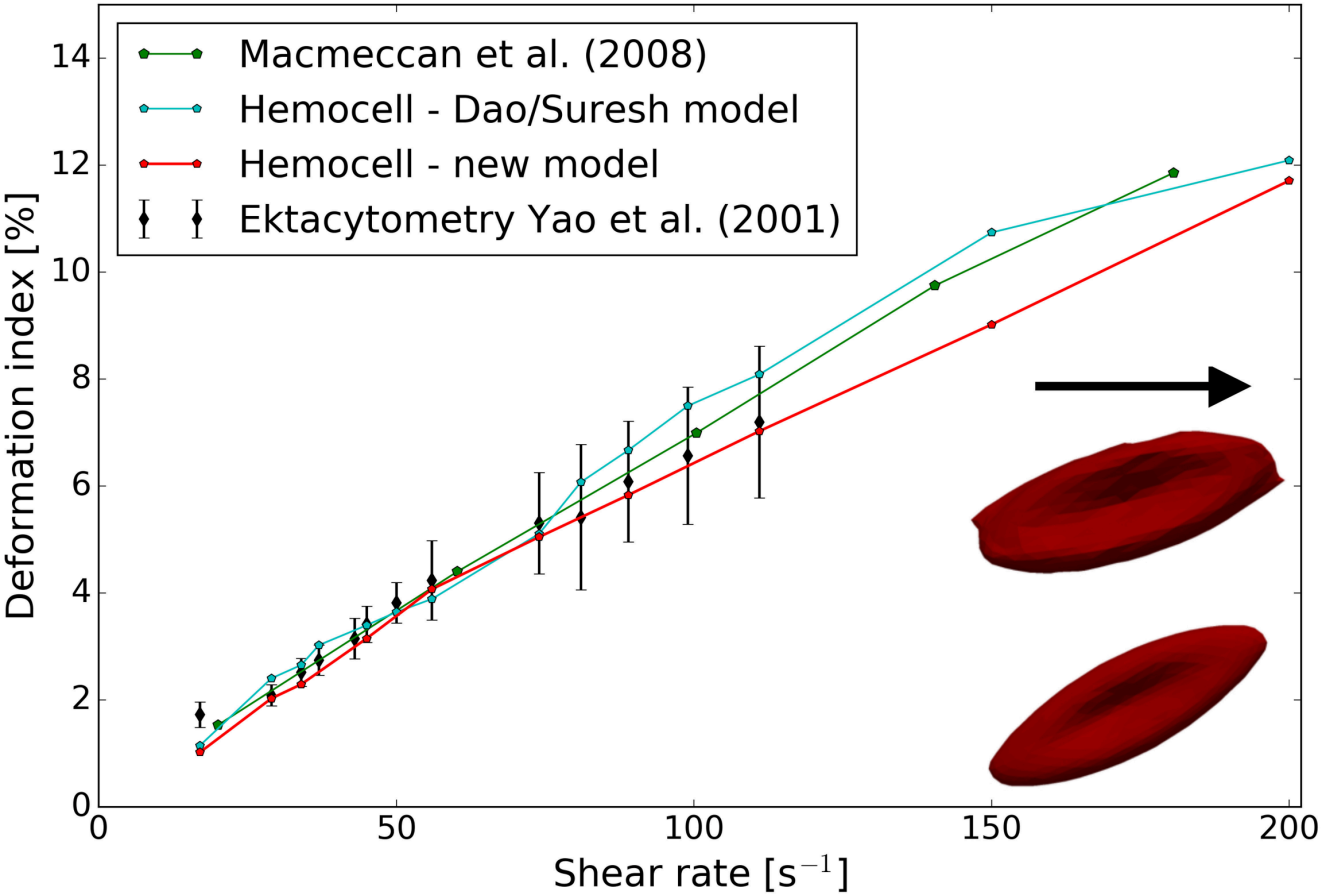
Results of the wheeler RBC shearing simulations. Both constitutive models show good agreement with the measurements. Inset image in the lower right corner: RBC shapes at an increased shear rate of 1, 500 *s*^−1^ for the two implemented material models—top, Dao/Suresh model; bottom, our model.

In the wheeler experiment performed by Yao et al. (2001) an RBC is positioned in shear flow such that the axis of symmetry of the cell lies in the plane of the shear and is perpendicular to the flow velocity. The deformation of the RBCs is then inferred from measuring its laser diffraction pattern in the flow. We numerically compute the behavior of a single RBC placed in pure shear flow with shear rates between 17 and 200 *s*^−1^, in accordance with the experiment. The deformation index of the RBC is defined as given in Yao et al. (2001):

$$DI=\frac{{\left(D_{max}/D_{0}\right)}^{2}-1}{{\left(D_{max}/D_{0}\right)}^{2}+1},$$

where *D*_0_ is the original diameter of the RBC (7.82 μ*m*) and *D*_*max*_ is the maximal diameter during the deformation at a constant shear rate value. The results are compared to the experimental results and to simulated results of MacMeccan et al. (2009) in Figure 5. Both material models give a deformation index that are in agreement with Yao's experiment and with the simulations of MacMeccan et al. (2009). It is important to point out that the numerical accuracy of this simulation is more sensitive to the fluid-structure coupling compared to the previous stretching scenario, therefore, a close match with the measurements implies an accurate coupling between the plasma flow and the cell membranes. Additionally, the numerical limit of shear rate is tested for both material models with this setting. In our implementation the new material model could resist higher sustained shear rates (${\dot{\gamma }}_{max}=2,500s^{-1}$) than the spectrin-link model (${\dot{\gamma }}_{max}=500s^{-1}$) before the RBC collapsed due to insufficient force response arising from numerical errors (for the onset of such an error see the inset image in Figure 5).

The fit to the experimental results yield κ_*l*_ = 15 *k*_*B*_*T*, κ_*a*_ = 5 *k*_*B*_*T*, and κ_*b*_ = 80 *k*_*B*_*T*. These values are used throughout this work for the new constitutive model. Evans (1983) measured the bending modulus to be in the order of 50 *k*_*B*_*T*, not far from our κ_*b*_. Additionally, with the selected κ_*a*_ value the local surface extensions under physiological flow conditions are smaller than the set limit of 30%, typically below 7%, which agrees with the literature (Fung, 1993). Please note that the selection of these parameters is not unique, other sets might exist that also fit the single-cell experimental results well with the proposed mechanical model. To infer further material characteristics of the model, we employed a simulation to deform a single hexagonal patch of the membrane (for an overview of the applied deformations see Figure 2II). The uniaxial stretching yields a surface Young modulus of *E*_*s*_ = 27.82 μ*N*/*m*. Assuming that the major deformation response arise from the membrane (the bilipid layer, and the spectrin, actin filaments) and its width in the range of 25 − 50 *nm* (Gov and Safran, 2005; Yoon et al., 2009), the typical Young modulus for small deformations *E* = 1 *kPa* of healthy RBCs (Maciaszek and Lykotrafitis, 2011) gives the surface tensile modulus of *E*_*s*_ = 25 − 50 μ*N*/*m*, in agreement with our results. The shear deformation of the patch yields μ = 10.87 μ*N*/*m*, close to the upper region of the measured ranges of 6 − 10 μ*N*/*m* (Mohandas and Evans, 1994; Park et al., 2011). Finally, area expansion gives a compression modulus of *K* = 21.88 μ*N*/*m* near the reported range of 18 − 20 μ*N*/*m* (Park et al., 2011). Assuming homogeneous isotropic linear behavior (that only holds for small deformations), the relation between the elastic constants yields a Poisson's ratio of 0.29, in the vicinity of the expected value of 1/3.

**Figure 6:**
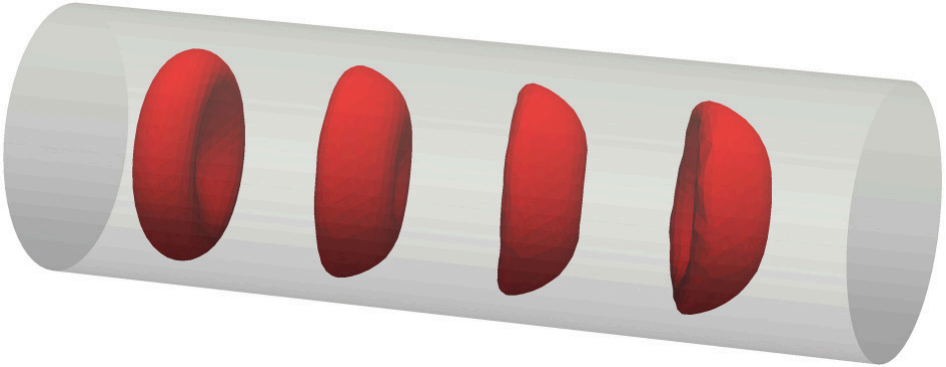
Transition of an RBC toward parachute shape while traveling in a micro-channel of *D* = 12 μ*m* diameter.

From the unique material properties an emergent ability of RBCs traveling in small, confined flows is their deformation to parachute-like shapes (Noguchi and Gompper, 2005). This behavior is necessary to pass small micro-capillaries of diameters below the diameter of the undeformed RBC (Tsukada et al., 2001). Figure 6 shows an example of a simulated RBC that deforms toward this shape in a tight channel computed with the new material model.

### 2.5. Generating cell initial conditions

**Figure 7:**
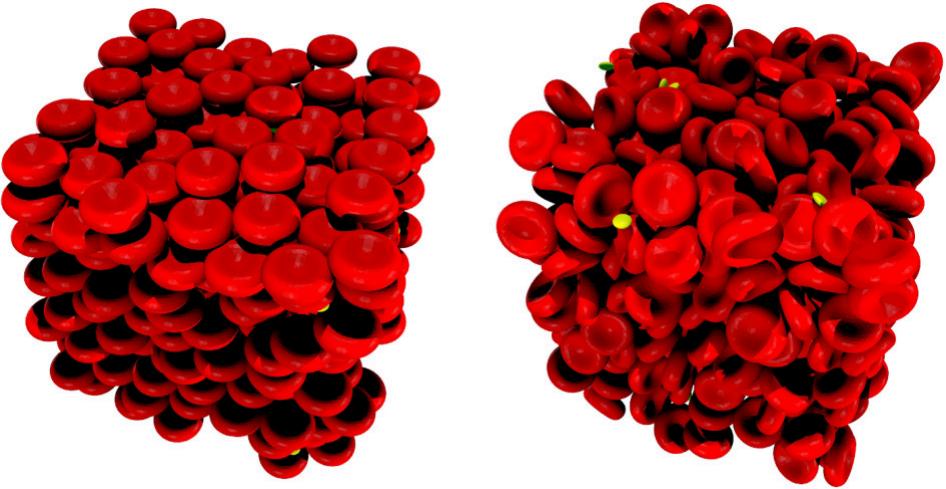
Randomized packing of a 50 μ*m*^3^ cubic domain with RBCs (red) and PLTs (yellow). **(Left)** random positions with fixed alignments. **(Right)** both positions and alignments are randomized.

An important component of simulating blood flows on a cellular level is the selection of initial conditions for the cells, such as position and orientation. These are far from trivial since, due to the biconcave shape of RBCs, their volume (≈ 71 μ*m*^3^) compared to the volume of their enclosing box (≈ 224 μ*m*^3^) is low. Using the densest possible packing along a regular grid thus yields a hematocit of 32% which is often inadequate as it does not reach the level of physiologic hematocrit of human blood. A further issue is the need for a randomized distribution to avoid initial artifacts originating from the regular positioning and orientations. To circumvent these difficulties an additional kinetic simulation was developed to compute realistic initial distributions even at high hematocrit values. Instead of the real biconcave shapes, the enclosing ellipsoid of the RBCs were used to execute a simple kinetic process for hard ellipsoid packing. The so-called the force-bias model (Mościński et al., 1989; Bargieł and Mościński, 1991; Bezrukov et al., 2002) was applied to these enclosing ellipsoids. The algorithm proceeds as follows. The positions of the center of the cells are randomly distributed in the simulation domain. Next, two scaling variables are defined for every cell type (e.g., RBC, platelet): *d*^*in*^ represents the possible largest scaling in the system without any overlap between the cells. While *d*^*out*^ is initially set so that the merged volume (counting overlapping volumes only once) of all the ellipsoids scaled with it equals the total volume of the enclosing ellipsoids corresponding to the desired hematocrit level. Then, a repulsive force is applied between overlapping ellipsoids, proportional to the volume of the overlapping regions:

$${\vec{F}}_{ij}=\delta_{ij}p_{ij}\frac{{\vec{r}}_{j}-{\vec{r}}_{i}}{\left|{\vec{r}}_{j}-{\vec{r}}_{i}\right|},$$

where **δ**_*ij*_ equals 1 if there is an overlap between particle *i* and *j* and 0 otherwise, while *p*_*ij*_ is a chosen potential function. In our case, the potential function was selected to be proportional to the overlapping volume of the *d*^*out*^ scaled particles. The positions are updated following Newtonian mechanics where mass is proportional to the particle scaling radius. This ensures that larger particles will move slower than smaller ones (i.e., an RBC will push away a platelet rather than the other way around). The rearrangement of the cells have a tendency of increasing *d*^*in*^. As a final step the size of *d*^*out*^ is reduced every iteration according to a chosen contraction rate τ. The computation stops when *d*^*out*^ ≤ *d*^*in*^ at which point the system is force-free, since there are no overlaps. Using this method, we were able to push the initial hematocrit value up to 46% covering the physiological range. Additionally, we can fix the orientation of the cells by only allowing translation of their center of mass during this computation, thus predefining the alignment of the particles. This is beneficial for initializing higher velocity flows where the cells are expected to be lined up with the bulk flow direction. Figure 7 presents two sample initial conditions generated with this method.

It is possible to initialize simulations of up to 10^6^ cells efficiently this way. These simulations are free from regular-grid positioning artifacts from the beginning, which in turn reduces the computational time significantly. Though the actual computational cost it saves varies by geometry, hematocrit, flow velocity, etc., in our simulations the warm-up phase needed to allow the initially regularly placed cells to arrange more realistically amounted to 10-30% of the total computational time, while with the randomized initialization this whole phase could be omitted.

## 3. Results

**Figure 8:**
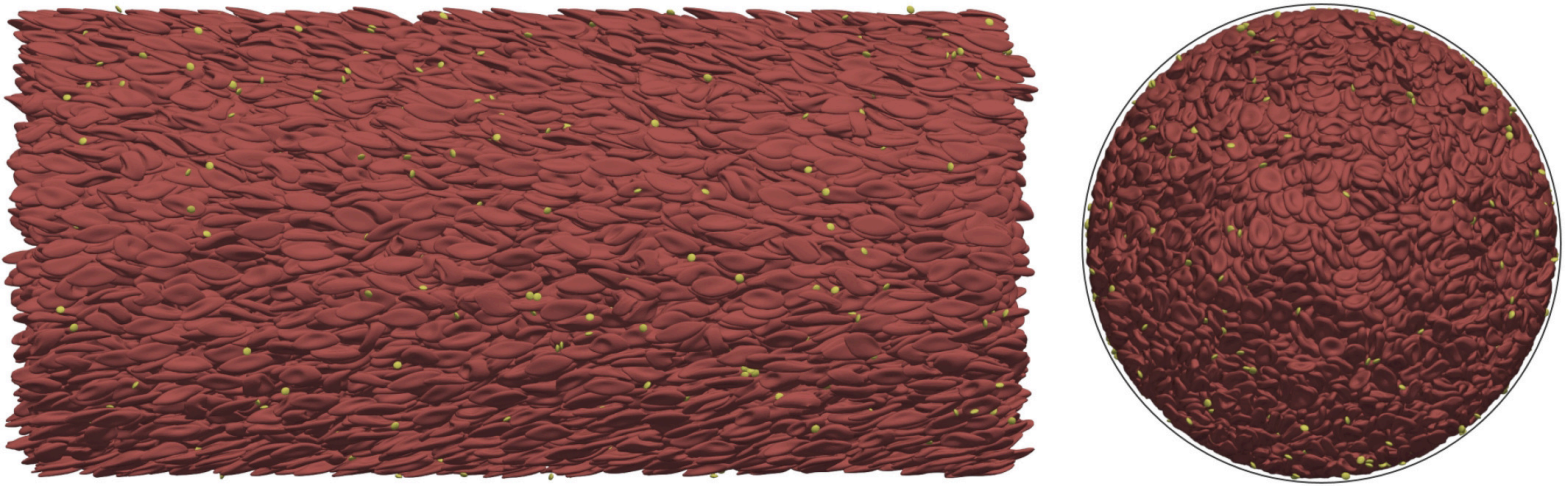
Blood flow simulation in a straight vessel section of *D* = 128 μ m diameter with a hematocrit value of 45% at an average velocity of 1.5 cm/s using our new material model. **(Left)** side view; **(Right)** 2D-projected axial view (the black outline shows the location of the vessel wall). The simulation of this case consists of ~2 × 10^4^ cells and it was computed on 512 cores.

Our ultimate goal of accurate mechanical modeling of cellular membranes in blood flows is to allow for the resolution of the collective transport dynamics and coupling this to relevant biochemical processes. In the following, these transport properties are explored using the new constitutive model in the cases of a straight vessel sections of varying diameters. A snapshot from the simulation of the *D* = 128 μ*m* case is visualized in Figure 8. The RBCs close to the wall experience much larger deformations than those in the center of the channel. In every simulation PLTs are present as well in a physiologic concentration (around 1/10th of the RBC cell count). Since the elastic response of the unactivated platelets are at least an order of magnitude stronger for small deformations than the response of RBCs (Haga et al., 1998), the platelets are simulated with the same constitutive model as RBCs, however, the constants κ_*l*_, κ_*a*_, κ_*b*_ are multiplied by 10. These simulations also benefit from the above mentioned randomized initialization of the cells.

**Figure 9:**
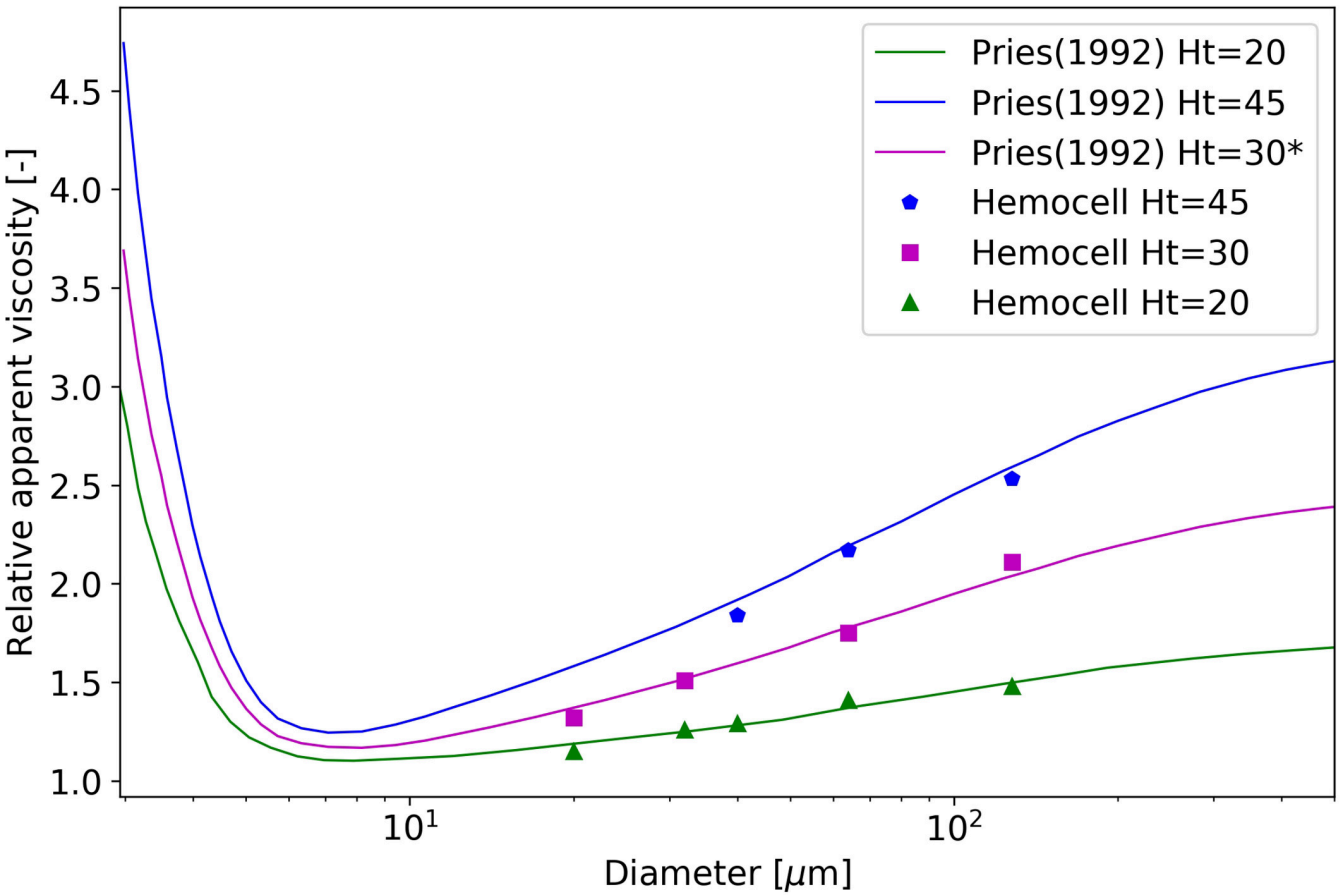
Relative apparent viscosity as a function of diameter at the hematocrit levels of 20, 30, and 45%. Please note that the curve of 30% originates from the fitted empirical formula of Pries et al. (1992).

The first fundamental transport property examined is the apparent viscosity. The results are compared to the experimental results collected by Pries et al. (1992). These experimental results are aggregated for hematocrit levels of 20, 45, and 60% after a correction for temperature and medium viscosity. Based on these data, an empirical formula is also derived in Pries et al. (1992) which was used in the current work to produce the expected results for the hematocrit level of 30%. It can be insightful to briefly overview the general measurement method of blood viscosity in experimental settings. The hematocrit level refers to the discharge hematocrit present in the blood tank, from where the flow is directed through a tube of various diameters driven by hydrostatic pressure. The relation of the pressure and the appearing average flow velocity in the tube defines the viscosity. This is taken into account in the current simulations by translating the discharge hematocrit values to hematocrit values actually present in the tube during the measurements by applying Equation (8) from (Pries et al., 1992). The simulations are initialized with zero velocity in the whole domain after which the flow is started up and driven by external body force. The results together with the experimental results are shown in Figure 9.

The results show good agreement with the measurements. For the simulations of *H* = 45% after the initialization the undeformed cells create large clusters. In the current work, the notation of an RBC cluster refers to a group of RBCs having at least a single membrane point in touch with another RBC of the same cluster. In the initial phase of the simulations the elastic effects of these RBC clusters are perceivable as the viscosity during the first few milliseconds increases quickly, well above the expected values. This is caused by the deformation of the cells residing inside these large and dense clusters and this behavior is one of the major components that leads to yield-stress. After a critical threshold in shape deformation they loose these stable structures and the viscosity quickly settles back to the expected level. For more details see Sections 3.1 and 3.2.

**Figure 10:**
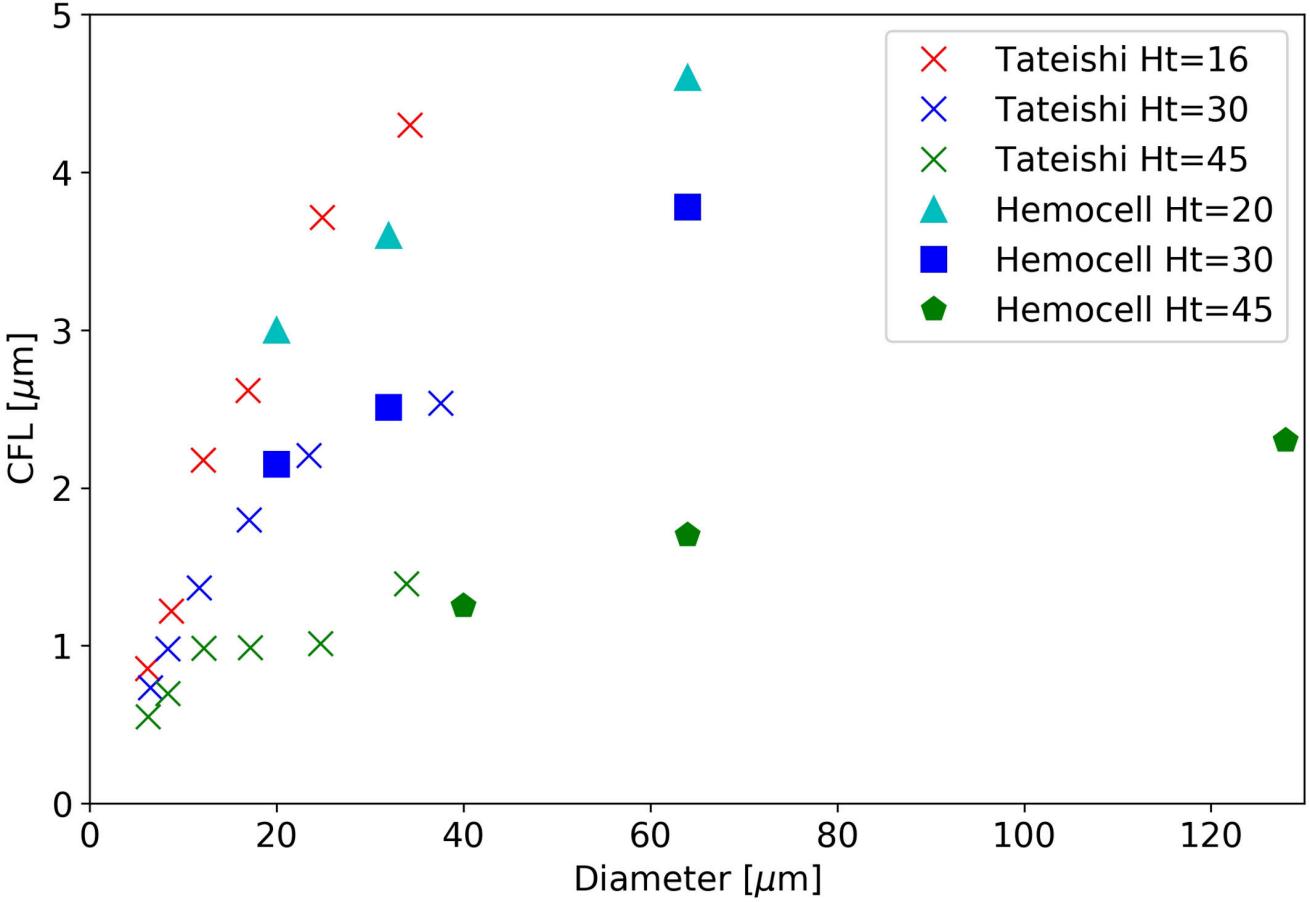
The width of the cell-free layer as a function of vessel diameter in a straight section.

Another distinctive feature of cellular suspension flows is the formation of a cell-free layer (CFL) close to the walls as a result of lift force acting on the cells. The width of the appearing cell-free zones are defined using the density distribution of cells. It is the distance from the wall at which point the density distribution averaged along the vessel section reaches 5%. The results are compared to the *in vitro* experiments at different hematocrit levels of Tateishi et al. (1994) in Figure 10.

While our simulated diameter range surpasses the bounds of the experimental range, the overlapping region shows good agreement for the hematocrit level of 30 and 45%. The level of 20% does not have a directly corresponding measurement, however, it is situated between between the experimental results of 16% and 30%, as expected. For a given diameter the CFL decreases with the increase of hematocrit as more RBCs are packed into the same domain volume.

**Figure 11:**
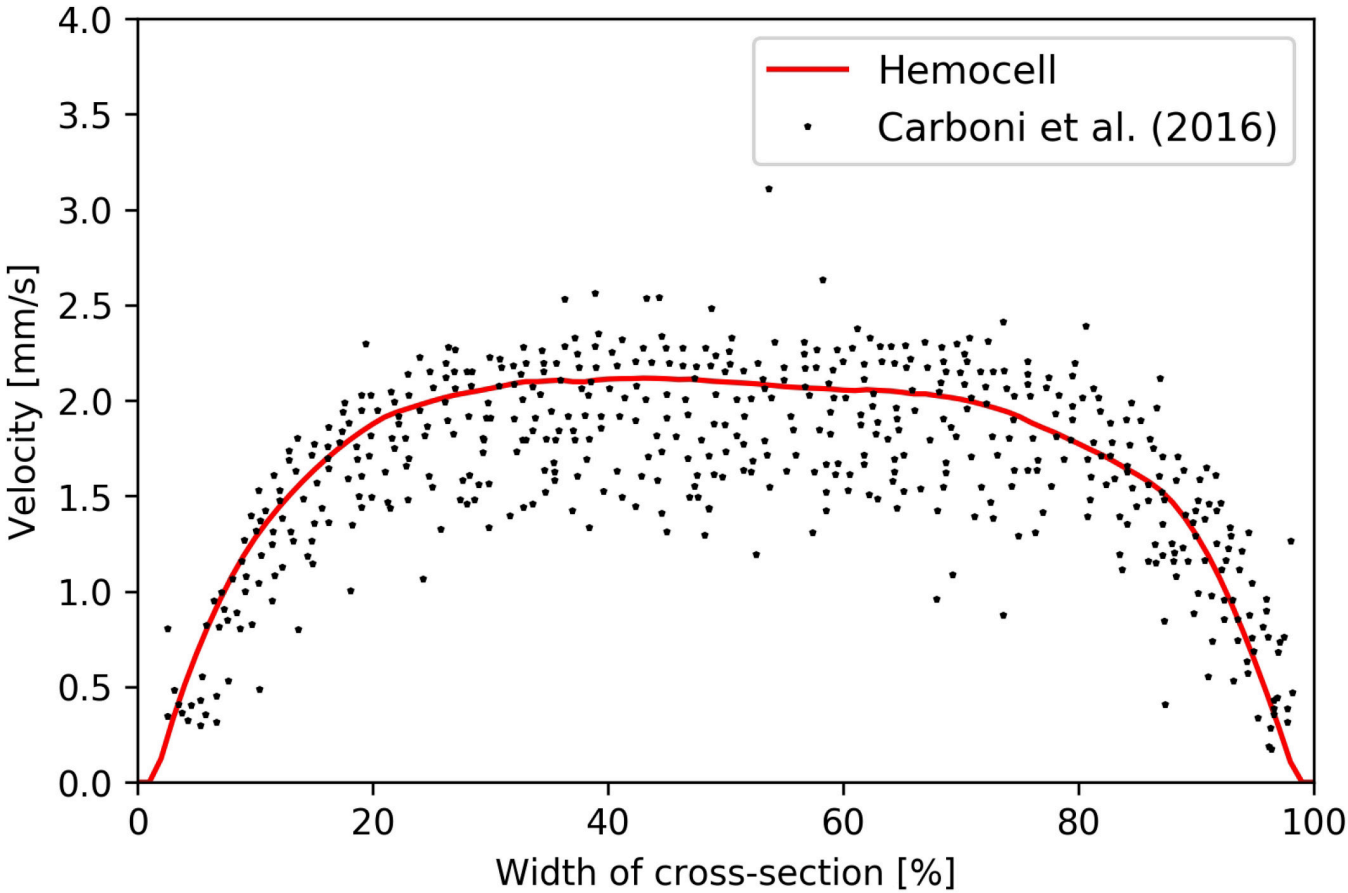
Velocity profile arising in Hemocell simulation (red line) and the particle tracking results of Carboni et al. (2016) (black dots).

Finally, to validate the flow profile in stationary flow a straight, rectangular channel was set up to recreate the flow environment of the experimental work of Carboni et al. (2016). The hematocrit level was set to 35%, and the driving body-force was calibrated to have a volumetric flow rate matching the experiment. In Figure 11, the velocity profile was compared to the profile obtained from the PIV measurements.

The simulated profile fits the measurement well and has the same plug-shape along with similar widths of high-shear regions at the sides of the channel.

### 3.1. Break-up of the RBC structures at increasing shear-rates

**Figure 12:**
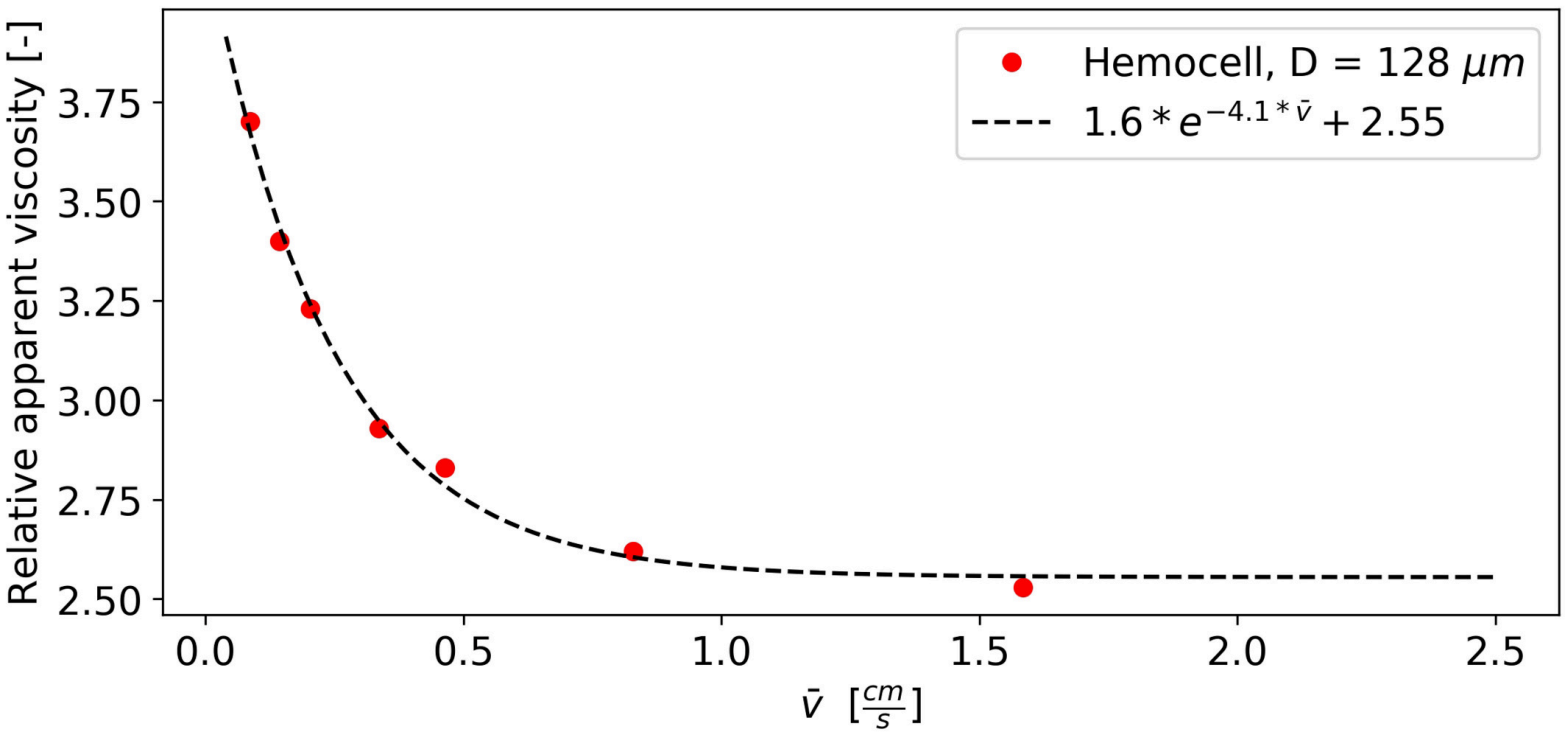
Viscosity drops as clusters are breaking up with the increase of flow velocity and shear.

It is a well-known phenomenon that toward low shear-rate values the viscosity of blood increases steeply (Chien, 1970). This is caused by the formation of dense clusters of RBCs including rouleaux structures. In our simulations, aggregation interactions between cells were not included, thus, these structures arise from the various alignments and high density of the cells. This effect was investigated in the case of the *D* = 128 μ*m* vessel section at *H* = 45%. The whole system is initialized to be still. Then, it is driven by a constant body force, and once the average velocity equilibrates (typically after a few hundred ms) the relative apparent viscosity is recorded. The shear is not constant along the radius of the vessel, however, for slow flows its local value scales approximately linearly with the average velocity. Figure 12 shows the relative apparent viscosity of the whole vessel section at low average velocities.

The relation appears to be logarithmic (see the fitted exponential decay), which is in agreement with the literature (Baskurt and Meiselman, 1997). Around the average velocity of 1 *cm*/*s* the apparent viscosity of the vessel section already settles suggesting that the majority of the RBC structures are gone. The further increase in velocity from this point on only results in a minor change of bulk viscosity.

### 3.2. Effects of the initial RBC deformation

**Figure 13:**
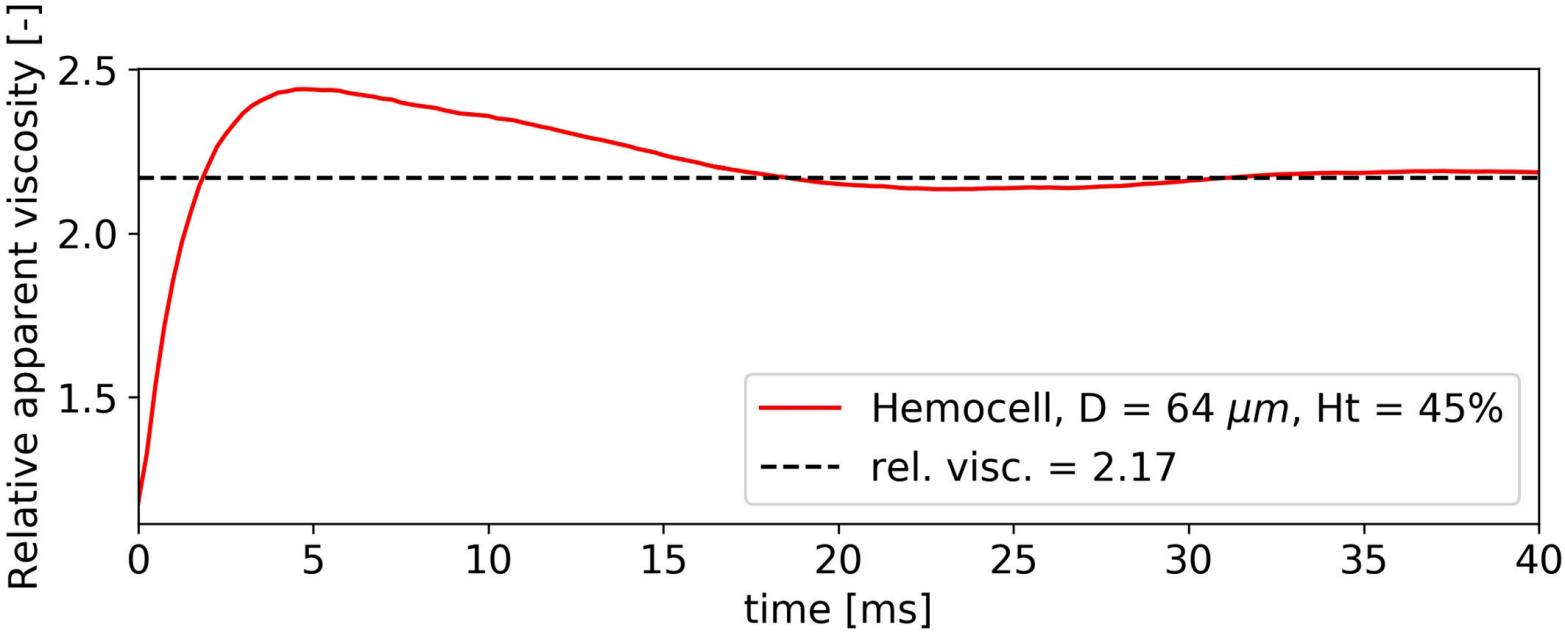
Elastic behavior of dense RBC clusters cause an initial viscosity peak.

Due to the elastic deformable nature of RBCs, blood can exhibit yield-stress behavior if the hematocrit level is high enough (Picart et al., 1998). In such a dense suspension of cells under low shear-stress the clusters can behave similarly to deformable solids. The relative positions of the RBCs within these clusters remain the same while they deform. At a critical stress value the force required to further deform the cells becomes larger than the force needed to separate them, thus breaking the structure. From that point blood transitions to fluid-like behavior. The stability of these clusters is dependent on several variables, for instance the level of hematocrit and the concentration of fibrinogen in blood plasma (Baskurt and Meiselman, 1997). However, a weaker yield-stress behavior still arises in the absence of fibrinogen (and other endogen proteins) at high hematocrit values (>30%) (Blackshear et al., 1983; Morris et al., 1989). This effect is perceivable during some phases of the simulations, such as the initial start up of the flows in our straight vessel sections. To investigate it, the plasma was brought up to the stationary velocity driven by external body force without any cells. This is necessary to separate the effects of initial cell deformations from the effects of initially driving the fluid up to the desired velocity. The velocity is set to a high enough value (e.g., $6\;\frac{mm}{s}$ for the *D* = 64 μ m, *H* = 45% case and $1.5\;\frac{\text{cm}}{\text{s}}$ for the *D* = 128 μ m, *H* = 45% case) for the large RBC structures to break. The undeformed cells with randomized positions and alignments are then placed into the flow while the driving force is kept constant. This moment is denoted as *t* = 0 s. Figure 13 shows the progression of the relative viscosity from this point in the case of *D* = 64 μ m, *H* = 45%.

During the first 3 ms the relative viscosity rises from the value of 1 steeply while the plasma flow slows down. At this stage, the RBCs do not flow but deform. The local velocity in the fluid corresponds to the deformation velocity of the cells. Around 4 ms, the relative viscosity reaches its peak value and the clusters start to break up, i.e., the relative positions of the RBCs start to change and the suspension no longer displays solid-like features. After this point blood quickly settles back to its stable final relative viscosity. The same viscosity pattern is observable for all simulations with *H* = 45% during the initial phase, however, for smaller diameters the phenomenon is less significant. It must be noted, however, that in our case both the surface and the cytoplasmic viscosity was the same as the plasma viscosity, while experimental results suggest higher values of 2−6 mPas for cytoplasma (Park et al., 2011) and 10^−10^ − 10^−9^ Ns/m for the bilipid membrane membrane (Waugh, 1982; Evans and Yeung, 1994). This difference is likely to have a strong effect on the characteristic times of cell deformation that is not investigated here.

## 4. Conclusions

The novel material model produces results in good agreement with several experiments targeting both single-cell mechanics and collective transport behavior. It also performs well for higher shear rate values where the other investigated model might fall short. It is capable of capturing the emerging solid-like behavior of dense RBC suspensions under low shear-rates. Furthermore, since our RBC material model is able to handle strong deformations coupling it with the LBM method for the plasma flow which operates at very small time-steps (in the order of 10^−8^*s* for the demonstrated flows) allows for small scale transient effects such as flow instabilities behind obstacles (e.g., stenosis or micro medical devices) to be simulated as well.

The framework itself is structured to be easy to extend with additional material models and cell types, e.g., white blood cells, and with other fields, such as concentrations of different chemical components as well as with new biophysical processes, for instance bond formations. The efficient highly parallel implementation is capable of handling large domain sizes, thus it is able to cover the range between cell-based micro-scale and macroscopic domains.

The demonstrated capabilities make this framework in combination with our constitutive model an ideal environment for exploring the transport effects of blood flows *in-silico*. It forms a solid ground for resolving accurate transport mechanics in vascular flows as a necessary component for modeling complex phenomena such as cell aggregation around micro-medical devices, thrombus formation and rheological response of diseases effecting RBC mechanical properties.

## Author contributions

GZ conceived the research, designed the model and wrote 50% the paper; BvR collected and analyzed the experimental data, validated the Dao/Suresh model in our implementation, revised the new material model, and wrote 50% of the paper; VA contributed to the technical realization of Hemocell and revised the final version of the paper; AH conceived and supervised the research, and revised the manuscript. All authors read and approved the final version of the manuscript.

### Conflict of interest statement

The authors declare that the research was conducted in the absence of any commercial or financial relationships that could be construed as a potential conflict of interest.

## Footnotes

^1^https://www.hemocell.eu/ (Accessed July 25, 2017).
